# Efficient data sampling scheme to reduce acquisition time in statistical ALCHEMI

**DOI:** 10.1093/jmicro/dfaf004

**Published:** 2025-01-13

**Authors:** Akimitsu Ishizuka, Masahiro Ohtsuka, Shunsuke Muto

**Affiliations:** Department of Materials Physics, Graduate School of Engineering, Nagoya University, Furo-cho, Chikusa-ku, Nagoya 464-8603, Japan; R&D, HREM Research Inc, 14-48 Matsukazedai, Higashimastuyama 355-0055, Japan; Department of Materials Physics, Graduate School of Engineering, Nagoya University, Furo-cho, Chikusa-ku, Nagoya 464-8603, Japan; Electron Nanoscopy Section, Advanced Measurement Technology Center, Institute of Materials and Systems for Sustainability, Nagoya University, Furo-cho, Chikusa-ku, Nagoya 464-8603, Japan; Department of Materials Physics, Graduate School of Engineering, Nagoya University, Furo-cho, Chikusa-ku, Nagoya 464-8603, Japan; Electron Nanoscopy Section, Advanced Measurement Technology Center, Institute of Materials and Systems for Sustainability, Nagoya University, Furo-cho, Chikusa-ku, Nagoya 464-8603, Japan

**Keywords:** ALCHEMI, fluorescent X-ray analysis, dopant site occupancy, wavelet transform, transmission electron microscopy, edge detection

## Abstract

The distribution of dopants in host crystals significantly influences the chemical and electronic properties of materials. Therefore, determining this distribution is crucial for optimizing material performance. The previously developed statistical ALCHEMI (St-ALCHEMI), an extension of the atom-location by channeling-enhanced microanalysis (ALCHEMI) technique, utilizes variations in electron channeling based on the beam direction relative to the crystal orientation. It statistically analyzes spectra collected across multiple beam directions. However, the total experimental time can be extensive, particularly for low dopant concentrations, where typical experiments can span several hours. In this study, we propose a scheme based on efficient sampling point selection that reduces the experimental time required while maintaining accuracy. Guidelines for selecting beam directions were derived from theoretical and experimental analyses of data redundancy. The strategies include choosing directions that exhibit greater variances in the host ionization channeling patterns and lower correlation coefficients between them. Additionally, an edge detection scheme using the dual tree complex wavelet transform, applied to electron channeling patterns, is proposed to significantly reduce measurement time. Our findings suggest that effective sampling can reduce experimental duration by at least two orders of magnitude without compromising accuracy. Implementing the proposed guidelines shortens total measurement times, minimizes electron irradiation damage and improves S/N ratio through extended data acquisition per tilt.

## Teaser

We propose an efficient scheme for sampling point selection that reduces the experimental time required while maintaining accuracy. This guideline was obtained based on theoretical and experimental data redundancy analyses. This reduces the total measurement times, minimizes electron irradiation damage, and improves S/N ratio through extended data acquisition per tilt.

## Introduction

Identifying the dopant distribution within a host crystal is crucial for improving semiconductor performance because the dopant distribution directly influences the macroscopic chemical and electronic characteristics of the material. A prevalent method for determining dopant distribution involves the direct examination of impurities using an aberration-corrected scanning transmission electron microscope (Ac-STEM) equipped with high-sensitivity detectors such as energy-dispersive X-ray (EDX) and electron energy-loss spectroscopy [1]. Although the STEM spectral-imaging technique can effectively reveal elemental distributions at atomic resolution, electrons exhibit wave-like behavior when propagating through solids. Consequently, even when targeted at sub-nanometer scales, the incident electrons tend to spread across adjacent atomic columns in thicker sample regions (exceeding 15–20 nm) [2]. Consequently, the precision of atomic column-by-column analysis is significantly influenced by the sample’s quality, encompassing factors like thickness, surface integrity and instrumental alignment issues such as the accuracy of sample orientation, lens astigmatism correction and sample drift.

Another technique to determine dopant distribution utilizes the fact that the propagation of incident electrons, known as channeling, varies with changes in the incident beam direction relative to the crystal orientation. This technique is called atom-location by channeling-enhanced microanalysis (ALCHEMI). ALCHEMI was first proposed by Taftø and Spence [3] using energy-dispersive X-ray spectroscopy (EDS), and by Taftø and Krivanek [4] using electron energy-loss spectroscopy. Although ALCHEMI requires data collected from only a few beam directions and involves relatively simple processing, its accuracy strongly depends on the signal-to-noise ratio (SNR) of the observed spectra. Consequently, long exposure times are typically required to reduce noise in the spectra.

To address the problems inherent to ALCHEMI, an alternativeapproach called statistical ALCHEMI (St-ALCHEMI) has been developed. St-ALCHEMI involves the collection of several dozen spectra while varying the incident beam directions, followed by statistical analysis of the observed spectra to estimate dopant occupancies [5].

St-ALCHEMI has been refined into more sophisticated techniques, such as high-angular resolution electron channeled X-ray spectroscopy (HARECXS) and high-angular resolution electron channeled electron spectroscopy (HARECES). In these methods, angular resolution is controlled using beam tilt deflectors with high precision [6–8]. The original HARECXS/HARECES concept relies on successive beam tilts along a systematic row of reflections to identify dopant planes among alternating stacked crystallographic planes, taking advantage of planar channeling phenomena. Subsequently, JEOL Inc. and FEI developed digital beam control systems for STEM, which facilitated HARECXS data acquisition. These systems involved an attachment-scanning image display (ASID) for JEOL STEMs and the open-source scripting program TIA running on FEI TEMs with attached detectors. These systems could efficiently tilt the incident beam directions in two dimensions, thereby extending the original HARECXS to a more generalized scheme for identifying dopant sites in crystals with any type of crystallographic symmetry [9–11]. However, probe movement due to lens aberrations in the electron illumination system restricts the minimum specimen area that can be analyzed to approximately 1 μm when the incident beam is tilted by a few degrees.

Recently, our group verified that St-ALCHEMI can be applied to areas of a few tens of nanometers by modifying a software tool called QED [12,13]. QED is a plug-in script for the Gatan Microscopy Suite that compensates for probe shifts caused by beam tilt [14]. This modification enables measurements over several tens of nanometers, although depending on the probe size, it can lead to a decrease in signal. Moreover, software-based beam control necessitates a longer total measurement time owing to the slower processing speed compared to that required by ASID hardware beam control.

Two-dimensional digital beam tilts have not only improved statistical accuracy by systematically increasing the number of sampling points but also expanded the applicability of the technique to more complex scenarios. These include cases where a dopant occupies multiple sites of the same host element, requiring a comparison between experimental and theoretical X-ray emission patterns [ionization channeling pattern (ICP)] [9,15]. Additionally, the ICP of a dopant generally resembles the host ICP of the dominant occupation site, enabling the preferential site of the dopant to be inferred through pattern recognition. However, a significant drawback is the lengthy total experimental time, which can extend to multiple hours depending on the dopant concentration. For instance, in a case where the dopant concentration is less than 1% and the number of beam tilts is set to a 32 × 32 grid, typical EDS acquisition times to obtain a sufficiently high SNR would range from 30 s to 60 s per beam tilt, resulting in a total experimental time of 5–10 h. Such prolonged experimental durations are not preferable as they pose potential electron irradiation damage risks and may require extended periods to achieve an improved SNR.

From a statistical perspective, a few tens of sampling points in a two-dimensional beam tilt over a few degrees are generally sufficient to obtain three significant figures for dopant site occupancies [16,17]. Therefore, effective selection of sampling points that avoids unnecessary data redundancy can generally reduce the total experimental time by at least two orders of magnitude.

In this report, we explore methods to reduce the total experimental time by selectively reducing the number of measurement points in an efficient manner, while preserving the level of accuracy achieved. The rest of the paper is structured as follows: We begin by mathematically analyzing data redundancy and propose a scheme for the efficient selection of sampling points. The proposed scheme is then validated using various datasets through simulations and experiments. Finally, we discuss the limitations of St-ALCHEMI and provide concluding remarks.

## Methods

### Evaluation of data redundancy

In St-ALCHEMI, we observe the characteristic X-ray emission counts *N_ij_* from each host element *i* (*i =*1, …, *K; K* is the number of host sites) and emission counts $N_j^x$ from substitutional impurities *x* at each incident beam direction *j*. We assume that the impurity counts are proportional to the host counts at the same site. Thus, the total impurity counts for *x* can be calculated as a linear combination of the host atom counts from all the sites:


(1) $$N_j^x = \mathop \sum \limits_i^K \alpha _i^x{N_{ij}} + {\beta ^x} + \varepsilon _j^x,$$

where $N_j^x,{N_{ij}} \ge 0$, ${\beta ^x}$ is an empirically introduced offset to improve the fit, and $\varepsilon _j^x$ is the regression error or residual. Assuming that the data are free from noise ($\varepsilon _j^x = 0$) and Eq. (1) holds strictly (i.e. the host and impurity elements have the same X-ray emission potential profile except for scaling factors, as discussed later), the minimum number of sampling points necessary should be *K *+ 1 for each incident beam direction. However, in actual experiments, the data always include some noise. The coefficients $\alpha _i^x$ and their uncertainties $\delta \alpha _i^x$ were determined using the least squares routines based on the experimental data (${N_{ij}}$ and $N_j^x$) observed at different incident beam directions *j*. The uncertainties of the impurity occupancies were determined from the accuracies of $\delta {\alpha _i}$ according to the error propagation formula.

For simplicity, let us consider the case in which a single dopant and two different host sites are involved. In such a case, Eq. (1) for the total number of tilts, *n*, reduces to the form:


(2) $$N_j^x = {\alpha _1}{N_{1j}} + {\alpha _2}{N_{2j}} + \beta + {\varepsilon _j},forj = 1,2,\ldots,n.$$

This can be expressed in the matrix form as


(3) $${\bf{\mathit{y = }}}A\alpha + \varepsilon ,$$

(4) $${\bf{\mathit{y}}} = \left( {\begin{array}{*{20}{c}}{N_1^x}\\\vdots \\{N_n^x}
\end{array}} \right),$$

(5) $$A = \left( {\begin{array}{*{20}{c}}
{{\boldsymbol{N}_1}}&{{\boldsymbol{N}_2}}&{\bf{\mathit{I}}}
\end{array}} \right),$$

(6) $${\boldsymbol{N}_i} = \left( {\begin{array}{*{20}{c}}{{N_{i1}}}\\\vdots \\
{{N_{in}}}\end{array}} \right),{\bf{\mathit{I}}} = \left( {\begin{array}{*{20}{c}}1\\\vdots \\1\end{array}} \right)\left( {i = 1,2} \right)$$

(7) $${\boldsymbol{{\alpha}}} = \left( {\begin{array}{*{20}{c}}{\begin{array}{*{20}{c}}{{\alpha _1}}\\{{\alpha _2}}\end{array}}\\\beta \end{array}} \right).$$

(8) $$\varepsilon = \left( {\begin{array}{*{20}{c}}{{\varepsilon _1}}\\\vdots \\
{{\varepsilon _n}}\end{array}} \right)$$

Eq. (3) is redundant for *n* > 3 and its compromise solution $\alpha $ is given by linear regression. The variance of ${\alpha _i}\left( {i = 1,2} \right)$, denoted ${\left( {\partial {\alpha _i}} \right)^2}$, is given by:


(9) $${\left( {\partial {\alpha _i}} \right)^2} = \frac{{\overline {\boldsymbol{N}_j^2} - {{\overline {{\boldsymbol{N}_j}} }^2}}}{\Delta }\frac{{{\sigma ^2}}}{n} = \frac{{{v_j}}}{{{v_1}{v_2} - v_{12}^2}}\frac{{{\sigma ^2}}}{n},\left( {j = 1,2,j \ne i} \right)$$

where


(10) $$\Delta = {v_1}{v_2} - v_{12}^2,$$

(11) $${v_i} = \frac{1}{n} \sum \left(\boldsymbol{N}_i - \overline{{\boldsymbol{N}_i}}\right)^2 = \overline{\boldsymbol{N}_i^2} - {\overline{{\boldsymbol{N}_i}} ^2},\left( {i = 1,2} \right)$$

(12) $${v_{ij}} = \frac{1}{n} \sum \left( {{\boldsymbol{N}_i} - \overline {{\boldsymbol{N}_i}} } \right)\left( {{\boldsymbol{N}_j} - \overline {{\boldsymbol{N}_j}} } \right) = \overline {{\boldsymbol{N}_i}{\boldsymbol{N}_j}} - \overline {{\boldsymbol{N}_i}} \cdot \overline {{\boldsymbol{N}_j}} ,\left( {i = 1,2} \right)$$

${\sigma ^2}$ is the variance of the estimated noise for the experimental data $y$, and $\bar x$ denotes the mean of ${x_i}$. Introducing a correlation coefficient ${C_{12}}$:


(13) $${C_{12}} = \frac{{{v_{12}}}}{{\sqrt {{v_1}} \sqrt {{v_2}} }},$$

we finally obtain the following formula for the variance ${\left( {\partial {\alpha _i}} \right)^2}$:


(14) $${\left( {\partial {\alpha _i}} \right)^2} = \frac{{{\sigma ^2}}}{{n{\nu _i}\left( {1 - C_{12}^2} \right)}}.$$

Equation (14) suggests that the uncertainty in the regression coefficient ${\alpha _i}$ is reduced by maximizing ${v_i}$ and minimizing $\left| {{C_{12}}} \right|$. Alternatively, selecting sampling points with intensities distributed over a wider range and ensuring minimal correlation between intensities from each host reduces uncertainties. For typical experimental conditions in the Eu-doped Ca_2_SnO_4_ case [9,17], we estimated $\left| {\partial {\alpha _i}/{\alpha _i}} \right| 0.3/\sqrt n $. This suggests that a few percent accuracy of ${\alpha _i}$ can be obtained with no more than 100 sampling points.

### Strategy for sampling point selection

In principle, beam directions can be optimized to minimize the number of measurements based on the aforementioned guidelines, provided the host ICP patterns are known. However, experimentally obtaining a set of host ICP patterns with sufficient SNR can take a long time. Therefore, a more efficient method for determining the measurement sampling points needs to be examined. The electron-channeling pattern (ECP), which can be experimentally obtained within minutes, even in software-driven mode using QED, presents a promising alternative. The original ALCHEMI procedure involves acquiring X-ray emissions under strong channeling conditions, where the Bloch wave interacts strongly with a particular set of atomic planes. The channeling orientation should be chosen such that the planes strongly interacting with the electron beam contain the impurity atom sites. Under the simplest two-beam diffraction condition, using a low-order diffraction vector perpendicular to the excited planes, the corresponding Bloch wave is either maximized or minimized on a plane of atoms, depending on whether the diffraction angle is slightly larger or smaller than the exact Bragg condition. Consequently, the bright-field image contrast changes significantly owing to the anomalous absorption effect (Borrmann effect), and the fluorescent X-ray from the atoms in that plane will be at its highest or lowest, respectively. This phenomenon provides a rough guideline for data sampling, as the X-ray intensities should be inversely correlated with the diffraction conditions that deviate slightly on either side of the exact Bragg condition.

The above principle cannot be directly extended to ECPs due to the complicated dynamical scattering effects. However, an ECP is likely to exhibit discontinuous contrast changes when the diffraction condition passes a Bragg condition as the beam direction continuously changes. This results in an inverse correlation between the corresponding ICPs. Therefore, ICP data should be preferentially selected from directions on either side of the edges where there is a significant variation in ECP intensity.

In this report, edge detection was performed using the two-dimensional dual-tree complex wavelet transform (DTCWT) [18], rather than the conventional FFT high-pass filter, to avoid image distortion near the image border caused by the periodic boundary condition. The ‘dualtree2’ and ‘idualtree2’ functions in MATLAB were applied to filter and reconstruct the image, with the ‘LowpassGain’ option set to zero. This approach enabled the reconstruction of images up to a predefined level of detail, depending on the resolution of the ECP pattern. A brief description of DTCWT is provided in Supplementary Information S1.

### Validation of the proposed method

#### Simulated data

In this section, we examine whether the proposed scheme ensures the required accuracy when the number of sampling points is reduced. We consider three representative cases, each requiring different strategies for selecting appropriate data points. Prior to the experiment, we used simulated ICPs to verify the sampling point selection strategy. The simulator outputs the inelastic-scattering cross-section, which corresponds to the excitation probability of the target element per unit dose. The simulation procedure is detailed in a review by Muto and Ohtsuka [9].

#### Case 1: Eu-doped Ca_2_SnO_4_

**Fig. 1.**
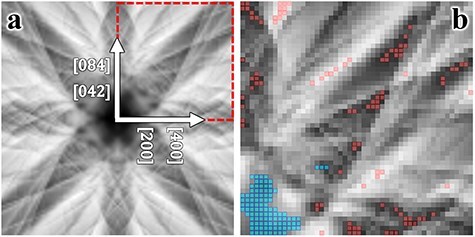
(a) Simulated ECP of Eu-doped Ca_2_SnO_4_ along the [012] zone axis, with a tilt grid of 101 × 101 spanning ±2 degrees. Red spots along the axes represent Bragg angles. (b) Red squares overlay the top 100 sampling points selected based on the absolute intensity within unique regions identified by edge detection. Cyan squares correspond to yellow dots on the right side of the vertical line in Fig. 2. Only one of the quadrants in (a) is shown, considering the ECP symmetry. Refer to the accompanying text for additional details.

In this study, we initially focus on Eu-doped Ca_2_SnO_4_, employing the St-ALCHEMI method [16,17]. Although the HARECX/ES technique does not inherently depend on the crystal structure details, we provide information on the arrangement of host atom sites, the crystal structure and the coordinates of elements in the unit cells in Supplementary data S2. Figure 1a displays the simulated ECP around the [012] zone axis, with Eu doping at the Ca and Sn sites at an identical concentration of 0.3%. For these simulations, the accelerating voltage was set to 200 keV, with the incident beam directions covering a square of $ \pm $2 degrees, mapped at 101 × 101 points. Notably, the variations in ECP and the corresponding ICP with sample thickness diminish significantly once the thickness exceeds 150 nm; therefore, we utilized a sample thickness of 150 nm. Edge detection was conducted using the wavelet reconstructed ECP at level 4, equivalent to a resolution of 16 pixels (Fig. 1b). The reconstructed image highlights positions with steep contrast gradients subtended by the positive and negative extrema, as shown in Supplementary Figs. S1 and S2 (Supplementary data S1). The host ICPs are possibly inversely correlated at these edge positions, with the magnitude of the extrema serving as a measure of the larger contrast changes. The detected edge positions were ranked in descending order based on the absolute values of the extrema; the top 100 positions (pixels) are highlighted in red in Fig. 1b.

The Eu site occupancies, based on the present sampling point selection, were calculated by applying the St-ALCHEMI method to the set of simulated ICPs. The results for the 100 and 200 observation directions selected through edge detection are presented in Table 1. To model the Eu ICP at low concentrations, Gaussian random noise with a standard deviation (SD) of 30% relative to the SD of the Eu ICP was added to the simulated data. The estimated occupancies for random sampling are also shown in the table for comparison. The uncertainties of the Sn site occupancy, estimated from the directions selected via edge detection, are marginally better than those determined from randomly selected observation directions, whereas the uncertainties of the Ca site occupancy are slightly worse. Therefore, the uncertainty in fractional occupancy at the Ca site, obtained through edge detection, is almost the same as that obtained from randomly selected directions, contrary to expectations. However, the uncertainty in fractional occupancy at the Sn site remains the same because there are only two possible dopant sites.

**Table 1.** Site occupancies and fractional occupancy of Eu, calculated using 100 and 200 beam directions selected by edge detection and random sampling

		Site occupancy (%)		Fractional occupancy (%)
Scheme	No. of sampling points	Ca site	Sn site	Ca site
Edge detection	100	0.30 ± 0.025	0.30 ± 0.027	67.2 ± 2.7
	200	0.31 ± 0.017	0.29 ± 0.019	67.7 ± 1.9
Random sampling	100	0.30 ± 0.018	0.29 ± 0.030	67.5 ± 2.7
	200	0.30 ± 0.013	0.30 ± 0.021	66.8 ± 1.9

**Fig. 2.**
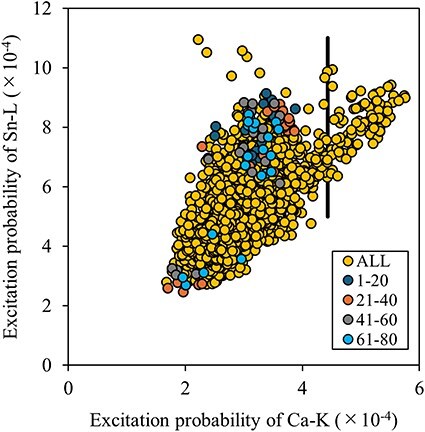
Distribution of excitation probabilities for the host elements in Eu-doped Ca_2_SnO_4_. The top 80 sampling points selected by edge detection are overlaid in the color-coded inset. Dots on the right side of the vertical solid line inset correspond to larger Ca-K emission intensities.

Table 1 suggests that the beam directions identified through the edge detection scheme are comparable to those selected randomly. To investigate this assertion, we plotted the relationship between the excitation probability of the host elements for each incident beam direction in Fig. 2. The excitation probability is calculated as the product of each atom’s inelastic scattering potential and the electron density, which varies with the incident beam direction. Essentially, the excitation probability reflects the ICP signal per unit dose, enabling visualization of the variation and correlation in the ICP signals across host elements. Each yellow dot in the figure represents a simulated incident beam direction, indicating the calculated excitation probabilities for both host elements. The top 80 incident beam directions chosen through edge detection are depicted in various color codes within the inset. Although Fig. 2 shows that edge detection successfully identified widely scattered beam directions, the excitation probabilities were found to be largely positively correlated. Therefore, the selected beam directions may not provide a significant advantage over random selections in reducing the number of measurement points required.

Table 2 summarizes the variances and correlation coefficients of host intensities for 100 observation directions chosen through edge detection and random selection. With edge detection, the variance of Sn was substantially larger than that obtained via random selection. Consequently, the accuracy of α_Sn_ and the dopant occupancy at the Sn site was marginally better than that achieved through random selection (see Table 1). In contrast, the variance for Ca-K emission intensities was smaller, and the accuracy of dopant occupancy at the Ca site was slightly worse than that obtained via random selection. This outcome verifies the effectiveness of the selection strategy, which prioritizes larger variances and smaller correlation coefficients.

**Table 2.** Variance ${v_i}$ (i = Ca, Sn) and correlation coefficients ${C_{Ca,Sn}}$ of the simulated excitation probabilities of the host atoms in Ca_2_SnO_4_ for 100 sampling directions by two selection schemes

	${\alpha _i}$ $\left( { \times {{10}^{ - 3}}} \right)$		${\left( {\partial {\alpha _i}} \right)^2}$ $\left( { \times {{10}^{ - 8}}} \right)$		${v_i}$ $\left( { \times {10}^{ - 8}} \right)$		
Scheme	Ca	Sn	Ca	Sn	Ca	Sn	${C_{Ca,Sn}}$
Edge	6.17	1.49	25.7	1.81	0.33	4.67	0.81
Random	6.15	1.47	13.7	2.36	0.42	2.36	0.68

In the present case, the Ca-K emission intensities were relatively weaker than those of Sn-L due to their respective inelastic scattering potentials, resulting in a suppression of the variance for Ca-K ICP to relatively lower values. Sampling points that exhibited higher Ca-K emission intensities, situated to the right of the vertical line in the inset of Fig. 2, can potentially increase the variance of the Ca intensity and decrease the correlation coefficient. These specific incident beam directions are marked by cyan dots in Fig. 1b. Notably, the majority of these cyan dots are clustered in the central area of the ECP. This pattern indicates that lighter elements cause less scattering of the incident electron beam, and the associated Bloch wave tends to propagate preferentially along planes containing lighter elements. The results are shown in Table 3.

**Table 3.** Occupancy, fractional occupancy and variance and correlation coefficients for 100 points selected according to the new guideline (a mixture of edge detection points and points near the ECP central region) Results obtained via random selection are shown for comparison

No. of points from central region (%)	Occupancy (%)		Fraction (%)	${v_i}$ $\left( \times {10}^{ - 8} \right)$		
	Ca site	Sn site	Ca site	Ca	Sn	${C_{Ca,Sn}}$
0	0.30 ± 0.025	0.30 ± 0.027	67.2 ± 2.7	0.33	4.67	0.81
10	0.30 ± 0.015	0.30 ± 0.022	67.3 ± 2.0	0.56	4.14	0.67
20	0.30 ± 0.012	0.31 ± 0.022	66.3 ± 1.9	0.71	3.47	0.59
30	0.30 ± 0.011	0.30 ± 0.023	66.9 ± 1.9	0.79	2.82	0.51
40	0.30 ± 0.010	0.31 ± 0.025	66.3 ± 2.0	0.85	2.34	0.44
50	0.30 ± 0.010	0.31 ± 0.024	66.4 ± 1.9	0.93	2.45	0.45
Random	0.30 ± 0.018	0.29 ± 0.030	67.5 ± 2.7	0.42	2.36	0.68

Increasing the proportion of points from the ECP central region resulted in higher ${v_{Ca}}$ and lower ${C_{Ca,Sn}}$, significantly improving the accuracy of Eu occupancy at the Ca site. Although the variance in Sn intensity decreased marginally, the accuracy of Eu occupancy at the Sn site improved owing to a reduced correlation coefficient. For Ca_2_SO_4_, optimal results were achieved when 20–30% of the incident beam directions were selected from the central region.

We then considered cases where the number of beam directions was reduced to 10. The points selected by edge detection in Fig. 2 are roughly divided into two groups: lower-left and upper-right, corresponding to positive and negative edge values, respectively. When edge selection is performed in descending order, the fraction of points selected from the lower-left corner can become too small, limiting the variation in sampling necessary for statistical accuracy, particularly when the total number of edge-selected points is small. Therefore, a better sampling guideline is to select an appropriate mix of edge detection points, ensuring that the lower-left group constitutes approximately 20–30% of the total edge selections. Table 4 presents the results for cases where the number of beam directions was reduced to 10, with the contribution of the lower-left group maintained at 25%. The theoretical accuracy is inversely proportional to the square root of the number of samples, and the results from random selection are consistent with this theory. However, the accuracy decreases more gradually when edge detection is used in combination with the addition of central directions. This occurs because edge detection selects directions that improve ${v_i}$ of the host intensity without inducing a significant change in ${C_{Ca,Sn}}$. Therefore, edge detection remains advantageous even when the number of sampling directions is reduced to 10.

**Table 4.** Variation in the accuracy as a function of the number of selection points determined via edge detection and random selection

		Occupancy (%)		Fraction (%)	${v_i}$ $\left( \times {10}^{ - 8} \right)$		
Scheme	No. of sampling points	Ca site	Sn site	Ca site	Ca	Sn	${C_{Ca,Sn}}$
Edge	10	0.31 ± 0.026	0.29 ± 0.057	68.5 ± 4.7	1.08	3.54	0.55
70%/center	30	0.30 ± 0.019	0.30 ± 0.034	66.6 ± 2.9	1.03	4.95	0.63
30%	100	0.30 ± 0.011	0.30 ± 0.020	66.6 ± 1.7	1	4.84	0.65
	10	0.30 ± 0.061	0.28 ± 0.099	67.5 ± 9.8	0.42	2.17	0.67
Random	30	0.30 ± 0.034	0.30 ± 0.055	66.5 ± 4.9	0.4	2.28	0.68
	100	0.30 ± 0.018	0.29 ± 0.030	67.3 ± 2.6	0.4	2.32	0.67

#### Case 2: Al-doped Y_2_Ti_2_O_7_

**Fig. 3.**
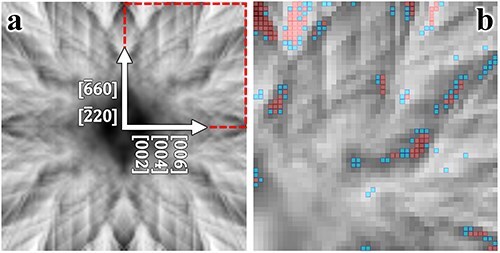
(a) Simulated ECP of Al-doped Y_2_Ti_2_O_7_ around the [110] zone axis, with a tilt grid of 101 × 101, which spans $ \pm 2$ degrees. Red spots on the axes denote Bragg angles. (b) The first and second best 100 beam directions determined by edge detection are marked as red and blue dots, respectively.

Al-doped Y_2_Ti_2_O_7_ was chosen as the second example. In this compound, Al is doped at 0.3% and 0.7% at the host Y and Ti sites, respectively. The difference in atomic numbers between the host elements in this case is not as pronounced as in the previous example; moreover, the atomic structures around Ti and Y are similar to each other [10] (see Supplementary data S2). Figure 3a shows the ECP obtained around the [110] zone axis, with all other conditions identical to those used to generate Fig. 1a. The edge detection results are displayed in Fig. 3b, where the top 200 beam directions are overlaid.

**Fig. 4.**
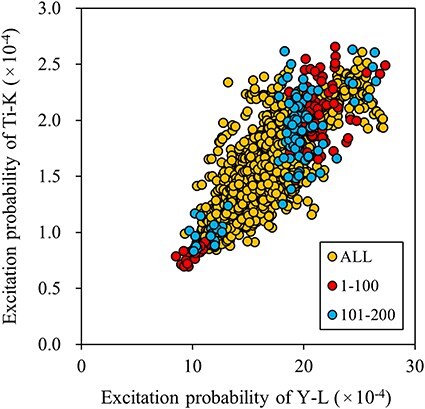
Distribution of excitation probabilities for the host elements (yellow dots) in Al-doped Y_2_Ti_2_O_7_. The top 200 sampling points from Fig. 4b are highlighted with red and blue dots.

Figure 4 shows the top 200 sampling beam directions overlaid on a 2D map of excitation probabilities for Y and Ti, showing a stronger positive correlation than that observed in Ca_2_SnO_4_. This suggests that reducing ${C_{Ti,Y}}$ via any sampling scheme is challenging.

Table 5 presents the resulting Al occupancies at the Y and Ti sites, as well as the fractional Al occupancy at the Y site, for 100 and 200 sampling points selected via edge detection. For comparison, the corresponding values obtained from random selections are also included. The comparison demonstrates that edge detection is an effective method for improving the overall measurement accuracy of dopant occupancies. The uncertainties of dopant occupancies at both Y and Ti sites were reduced by approximately 20% compared to those obtained through random selection, and the uncertainty of fractional occupancy at the Y site decreased by approximately 20%. This improvement can be attributed to significantly larger ${v_i}$ values in edge detection compared to those from random selection. However, the ${C_{Ti,Y}}$ values were slightly lower for random sampling, which made the accuracy improvement from the edge detection scheme less pronounced.

**Table 5.** Occupancies, fractional occupancy of Al, (i = Y or Ti) and of host element signals for Al-doped Y_2_Ti_2_O_7_ obtained via edge detection and random selection using 100 and 200 sampling points

		Occupancy (%)		Fraction (%)	${v_i}$		
Scheme	No. of sampling points	Y site	Ti site	Y site	Y ($ \times 10^{ - 7}$)	Ti ($ \times {10}^{- 9}$)	${C_{Ti,Y}}$
Edge	100	0.32 ± 0.051	0.71 ± 0.051	30.9 ± 3.8	2.65	3.46	0.92
	200	0.33 ± 0.037	0.69 ± 0.037	32.1 ± 2.7	2.48	3.25	0.91
Random	100	0.34 ± 0.060	0.70 ± 0.059	32.4 ± 4.4	1.06	1.42	0.83
	200	0.33 ± 0.042	0.70 ± 0.041	32.2 ± 3.1	1.11	1.48	0.83

Similar to the case of Ca_2_SnO_4_, we investigated whether sampling beam directions from the central ECP region could enhance accuracy. However, no appreciable improvement was observed, as the ${v_i}$ and ${C_{Ti,Y}}$ values did not change significantly (see Supplementary data S2).

We then examined whether edge detection selection remains effective when the number of measurement directions is reduced to 10 in Y_2_Ti_2_O_7_, while adhering to the new selection guidelines detailed in the previous subsection (i.e. ensuring that the lower-left group constitutes approximately 25% of the total edge selections). Table 6 presents the results obtained via edge detection and random selection while varying the number of measurement directions. The ${v_i}$ values of the host intensity obtained through edge detection increased as the number of measurement directions decreased, while the effect on the correlation coefficient was negligible. This improvement in host intensity ${v_i}$ was achieved by eliminating beam directions with lower absolute edge values. Conversely, in the case of random selection, the ${v_i}$ values of the host intensities and the ${C_{Ti,Y}}$ values did not change significantly with an increase in the number of beams. Therefore, the accuracy of random selection is inversely proportional to the square root of the number of measurements. However, the deterioration in accuracy with edge detection was lower than with random selection, even when the number of samples was reduced to 10.

**Table 6.** Comparison of accuracy as a function of the number of selected points for edge detection and random selection

		Occupancy (%)		Fraction (%)	${v_i}$		
Scheme	No. of points	Y site	Ti site	Y site	Y ($ \times {10}^{{ - 7}}$)	Ti ($ \times {10}^{{ - 9}}$)	${C_{Ti,Y}}$
Edge detection	10	0.37 ± 0.115	0.66 ± 0.110	35.7 ± 8.3	3.25	4.52	0.9
	30	0.36 ± 0.080	0.67 ± 0.077	35.1 ± 5.8	3.2	4.37	0.92
	100	0.32 ± 0.052	0.70 ± 0.051	31.3 ± 3.8	2.65	3.46	0.92
	10	0.31 ± 0.200	0.74 ± 0.200	29.2 ± 16.0	1.03	1.27	0.82
Random	30	0.32 ± 0.110	0.71 ± 0.107	31.1 ± 8.2	1.06	1.42	0.82
	100	0.34 ± 0.060	0.70 ± 0.059	32.4 ± 4.4	1.06	1.42	0.83

#### Case 3: Si-doped GaAs

**Fig. 5.**
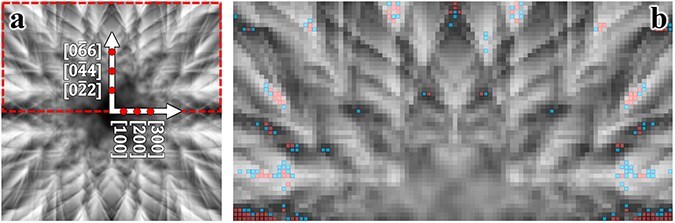
(a) Simulated ECP of Si-doped GaAs along the [011] zone axis, with a tilt grid of 101 × 101 spanning ±2 degrees. Red spots on the axes indicate Bragg angles. (b) The top 200 directions identified by edge detection are shown over areas of unique symmetry. Red and cyan dots represent the first and second set of 100 directions, respectively. Note: The crystal polarity in the horizontal direction is barely recognizable in the ECP.

**Fig. 6.**
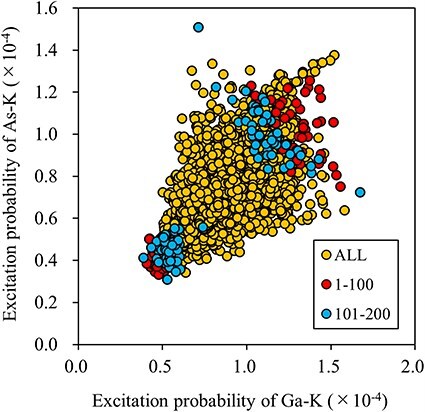
Distribution of excitation probabilities of host elements selected by edge detection for Si-doped GaAs (yellow dots). The first and second best 100 beam directions marked in Fig. 5b are respectively plotted as red and blue dots.

We consider Si-doped GaAs [011] as the final test case. The host crystal structure in Si-doped GaAs exhibits uniaxial asymmetry, or polarity, along the <100> direction. In previous studies on Si-doped GaAs [19], it was found that a very small amount of Si can be incorporated into the GaAs matrix via nearly equimolar substitution at the Ga and As sites, even when Si was doped to its maximum limit. In the model simulation, Si was thus doped at 0.08% and 0.07% at the Ga and As sites, respectively; the site occupancies were deliberately adjusted to deviate from equimolar substitution to facilitate the evaluation of accuracies at the respective sites. Figure 5a shows the simulated ECP for Si-doped GaAs. Edge detection was then performed using the same method described in the previous two cases. The top 200 beam directions selected by edge detection were plotted on the reconstructed ECP image and the excitation probability space of the host elements, as shown in Fig. 5b and Fig. 6, respectively.

Figure 6 shows that the excitation probabilities of the host elements are relatively weakly correlated compared to the stronger positive correlations observed in Ca_2_SnO_4_ and Y_2_T_2_O_7_. Given that Ga and As are close in the periodic table, their elastic and inelastic scattering powers are expected to be similar. Therefore, the top-left points in Fig. 6, selected by edge detection with negative values, are not biased toward the stronger element, unlike in the cases of Ca_2_SnO_4_ or Y_2_T_2_O_7_ (see Figs. 2 and 4). Therefore, edge detection successfully sampled points leading to larger ${v_i}$and smaller ${C_{Ga,As}}$ values, as listed in Table 7. The accuracy of the occupancies and the fraction of Si were approximately 15–20% better than those obtained via random selection.

**Table 7.** Occupancies, fractional occupancy of Si and variance and correlation coefficients of host element signals, calculated using 100 and 200 beam directions selected via edge detection or random selection

		Occupancy (%)		Fraction (%)	${v_i}$ $\left( \times {{10}^{ - 9}} \right)$		
Scheme	No. of points	Ga site	As site	Ga site	Ga	As	${C_{Ga,As}}$
Edge	100	0.077 ± 0.0056	0.070 ± 0.0060	52.2 ± 2.8	1.69	1.09	0.9
	200	0.077 ± 0.0038	0.072 ± 0.0039	51.9 ± 1.8	1.36	0.98	0.86
Random	100	0.081 ± 0.0071	0.071 ± 0.0069	53.2 ± 3.3	0.38	0.3	0.66
	200	0.080 ± 0.0051	0.070 ± 0.0049	53.4 ± 2.3	0.38	0.3	0.67

The host Ga and As ICP intensities in the $\left[ {100} \right]$ direction (the horizontal direction of Fig. 5) tend to vary in a negatively correlated manner, owing to the crystal polarity in this direction. The corresponding Bloch wave symmetry is interchanged upon passing through the exact Bragg condition, as described in the ‘Strategy for sampling point selection’. Therefore, preferential sampling along the polarity direction can serve as an alternative to ensure high accuracy.

Table 8 presents the occupancies, fractional occupancy of Si, variances and correlation coefficients of the host element signals analyzed from the ICPs sampled along the $\left[ {100} \right]$ direction using the [011] zone axis. The analysis includes two scenarios: one with 100 beam directions sampled at equal intervals, and another excluding 20 points near the [011] zone axis from the first. The latter scenario resulted in smaller variances and correlation coefficients compared to those obtained via random selection. When comparing the occupancy accuracies across different sampling methods, both random sampling and even sampling without excluding the central area showed similar and optimal accuracies. However, even sampling that included the central area produced unusual occupancies. This issue is further examined in the ‘Discussion’ section.

**Table 8.** Occupancies, fractional occupancy of Si and variance and correlation coefficients of host element signals, calculated using 80 and 100 beam directions selected evenly along the $\left[ {100} \right]$ direction. Sampling 80 directions excluded the points near the zone axis The results obtained from sampling 80 points via edge detection and random sampling are also tabulated

		Occupancy (%)		Fraction (%)	${v_i}$ $\left( \times {{10}^{ - 9}} \right)$		
Scheme	Points	Ga site	As site	Ga site	Ga	As	${C_{Ga,As}}$
w center	100	0.064 ± 0.0079	0.040 ± 0.0076	61.5 ± 5.3	0.29	0.23	0.23
w/o center	80	0.078 ± 0.0063	0.071 ± 0.0063	52.5 ± 3.0	0.26	0.19	0.15
edge	80	0.074 ± 0.0069	0.070 ± 0.0071	51.4 ± 3.4	1.69	1.17	0.92
random	80	0.081 ± 0.0081	0.071 ± 0.0079	53.0 ± 3.8	0.38	0.3	0.67

### Experimental verification

#### Tilt pattern implementation in QED

We modified the QED to enable automatic acquisition of a series of spectra for a set of predefined beam tilt directions from a small specimen area. A list of functions and sample scripts is provided in Supplementary data S3.

In the following experiments, we used a JEOL JEM-2100 STEM (operated at 200 keV) equipped with a silicon drift detector, Dry SD60GV (sensor size 60 mm^2^) (JEOL, Japan). The smallest condenser aperture (10 μmϕ) with α-selector ‘1’ was used to produce a quasi-parallel beam with a convergence semi-angle of 1.0 mrad. Using the largest spot size, ‘1,’ the probe size was approximately 200 nm in diameter. Illumination aberrations were corrected using QED, ensuring that the probe position remained almost stationary, thereby enabling data acquisition from a specimen measuring approximately 200 nm in diameter.

#### Eu-doped Ca_2_SnO_4_

**Fig. 7.**
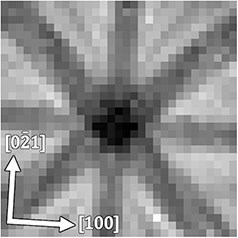
Experimental ECP of 20 at% Eu-doped Ca_2_SnO_4_ was used for edge detection.

We analyzed the site occupancies in 20% Eu-doped Ca_2_SnO_4_ following the current guidelines, with sample preparation details described in previous studies [16,17]. The sample was initially thinned using an FIB-SEM, Ethos NX5000 (Hitachi High-Tech, Japan), employing a 30 keV Ga ion beam. Final thinning was achieved with a 5 keV beam to remove surface damage, resulting in a sample thickness of 150 nm. Figure 7 shows the ECP obtained experimentally from 32 × 32 beam directions over an angular range of ±2 degrees along both the x and y axes. The exposure time for each direction was set at 1 ms to ensure an ECP with sufficient SNR, totaling approximately 4 min for the entire exposure. The majority of this time was dedicated to controlling the beam.

Using this ECP, edge detection was performed in a manner similar to that used in the simulation. A total of 100 beam directions were selected, with 70 chosen based on edge detection and 30 added near the zone axis. The spectra for each beam direction were automatically acquired, controlled by a script via QED, using a list of 100 beam tilt angles along the *x* and *y* directions. The exposure time for each beam direction was 20 s, resulting in a total experimental time of approximately 40 min to measure 100 points. Notably, measuring 31 × 31 beam directions requires approximately 4 h.

The occupancies of Eu at the Ca and Sn sites were estimated to be 6.8 ± 0.26% and 14.4 ± 0.85%, respectively, with a fractional occupancy at the Ca site of 48.5 ± 1.77%. These uncertainties are lower than those observed in the simulation, likely due to a better SNR, but are comparable to previous results obtained via HARECXS using QED [14].

Table 9 also presents the results when the top 10 and 30 points were selected using the same sampling scheme. As the number of selected points decreased, the variance of the host intensity increased, while changes in the correlation coefficient were not significant. Consequently, the increase in uncertainty was less than the inverse of the square root of the observations. Therefore, the edge detection method remains effective, even when the number of selection points in the experiment is reduced.

**Table 9.** Occupancies, fractional occupancy of Eu and variance and correlation coefficients of host element signals in 20 at% Eu-doped Ca_2_SnO_4_ They were calculated using 10, 30 and 100 beam directions of experimental data

	Occupancy (%)		Fraction (%)	${v_i}$ $\left( \times {{10}^7} \right)$		
No. of points	Ca site	Sn site	Ca site	Ca	Sn	${C_{Ca,Sn}}$
10	6.6 ± 0.50	15.5 ± 1.80	46.2 ± 3.48	5.42	4.63	0.88
30	6.7 ± 0.36	14.6 ± 1.23	47.8 ± 2.52	4.24	4.24	0.88
100	6.8 ± 0.26	14.4 ± 0.85	48.5 ± 1.77	3.11	3.42	0.87

#### Si-doped GaAs

**Fig. 8.**
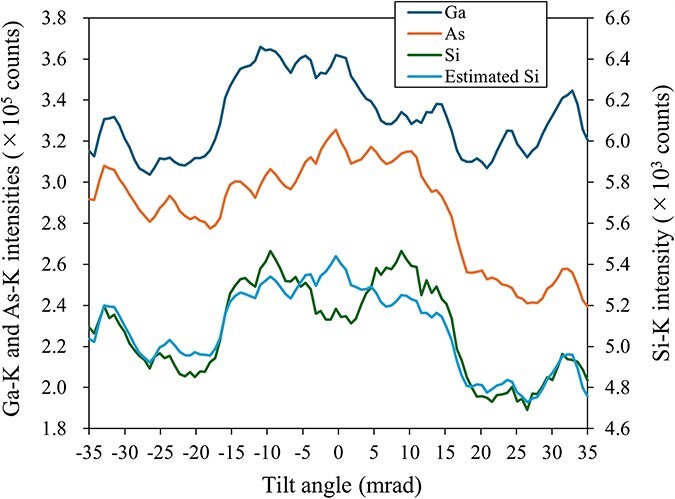
Observed ICPs of Si-doped GaAs along the polarity direction of the [011] zone. The estimated Si ICP with a linear combination of host ICPs is also shown.

In a separate experiment, we analyzed data sampled along the 〈100〉 polarity direction. The preparation involved dimpling the sample before ion polishing it with a 4 keV Ar ion beam, followed by another polishing with a 1 keV beam to minimize damage. This process helps maintain a thickness of 150 nm. A low-resolution ECP was sufficient to ascertain both the polarity direction and the orientation of the zone axis. We acquired the ECP from 32 × 32 beam directions, spanning ±2 degrees on both the *x* and *y* axes. The total measurement time for ECP acquisition was approximately 4 min, with an exposure time of 1 ms for each beam direction. The 〈100〉 polarity direction was determined from the obtained ECP, and 100 beam directions were selected by equally dividing the line passing through the zone axis along this polarity direction. The exposure time for each spectrum acquisition was 30 s, resulting in a total experimental time of approximately 50 min. Figure 8 shows the ICPs of the host and dopant elements obtained from this experiment.

Expressing the large dip in the Si ICP near the zone axis as a linear combination of the host ICPs proved challenging. When the central part was excluded, as discussed in the simulation section, the estimated Si occupancies at the Ga and As sites were 0.080 ± 0.006% and 0.070 ± 0.004%, respectively, with a fractional occupancy at the Ga site of 53.15 ± 2.15%. This demonstrates that the accuracy typically achieved with ALCHEMI can be obtained from a much smaller region, well below the sub-micron scale.

## Discussion: limitations of the St-ALCHEMI assumption

**Fig. 9.**
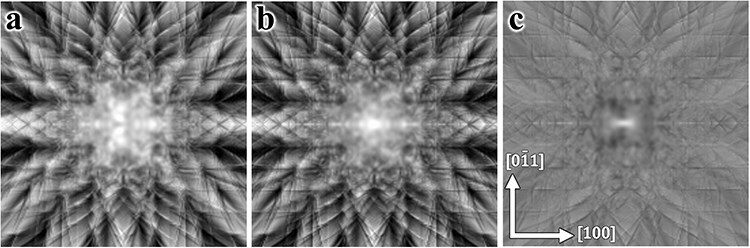
Simulated Si-K ICP (a) and resulting fit obtained via a linear combination of host ICPs to (a) (b) for Si-doped GaAs. (c) Difference between (a) and (b).

For Si-doped GaAs, Table 8 and Fig. 8 indicate that expressing the Si signal as a linear combination of host element signals is difficult when the incident beam direction is close to the zone axis. We examined the limitations of St-ALCHEMI using the linear combination ICP analysis (Eqs. (1) and (2)). Figure 9a and b shows the simulated Si-K ICP and the result fitted with the host ICPs, respectively, under conditions identical to those described in a previous subsection. Figure 9c shows the difference between Fig. 9a and b. The discrepancy between the simulated and fitted ICPs is significant near the center, particularly along the polarity direction, as illustrated in Fig. 9c. This indicates that the observed ICP cannot be accurately approximated by a linear combination of the host ICPs around the zone axis, explaining the anomalous values of the occupancies and fractional occupancy when beam directions near the zone axis along the polarity direction are included.

**Fig. 10.**
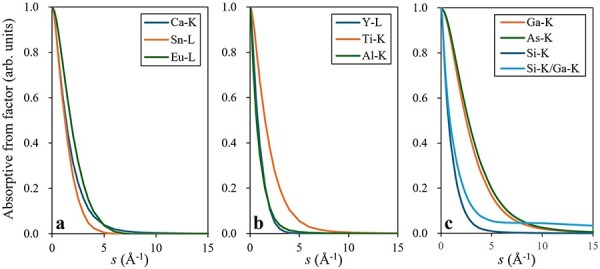
Absorptive form factors of (a) Ca-K, Sn-L and Eu-L for Eu-doped Ca_2_SnO_4_, (b) Ti-K, Y-L and Al-K for Al-doped Y_2_Ti_2_O_7_ and (c) Ga-K, As-K and Si-K for Si-doped GaAs. The ratio of Si to Ga is also shown in (c). Each AFF is normalized by the value at *s* = 0.

The excitation probability of an inelastic event (proportional to the fluorescent X-ray emission) for each element is determined by the product of the incident beam electron density distribution in the sample and the absorption potential of each element, which is calculated from the absorptive form factor (AFF) [21,22]. Figure 10a–c displays the AFFs of the host and dopant elements as a function of ***s*** (*s* = $sin\theta /\lambda $; *θ*: scattering angle; *λ*: electron wavelength), used in the simulations for Eu-doped Ca_2_SnO_4_, Al-doped Y_2_Ti_2_O_7_ and Si-doped GaAs, respectively, with each AFF normalized to its value at s = 0. These figures show that the distributions of the Ca K-edges, Sn L-edge and Eu L-edge are approximately similar, while the K-edge of Si is approximately three times narrower than the K-edges of Ga and As. The narrower AFF in reciprocal space indicates that the absorption potential in real space extends farther from the atomic center.

However, the Si AFF was nearly proportional to the Ga AFF at high angles (***s*** > 5 Å^−1^), as indicated by the ratio of Si-K/Ga-K (Fig. 10c).

The ECPs and ICPs in this study were acquired around low-order zone axes. Although this is not a strict requirement for St-ALCHEMI, and arbitrary crystal orientations can be used based on the same criteria, ECPs/ICPs generally show less distinct contrast for higher-order crystal orientations with smaller intensity variances. Therefore, measurements near the lower-order zone axes are preferable to ensure more accurate site occupancies.

## Concluding remarks

We previously developed a two-dimensional HARECXS method for analyzing dopant site occupancy from areas as small as 30 nm in diameter, based on a statistical ALCHEMI method using computer-controlled digital beam tilts. This method improves statistical accuracy and can be extended to more complex cases. In this study, we theoretically and experimentally explored strategies to reduce total experimental time by selectively reducing the number of measurement points based on data redundancy analysis. In particular, we ensured that the achieved level of accuracy was maintained, thereby addressing a significant drawback of the method: lengthy experimental times. The guidelines for effective selection of sampling points (beam directions) can be summarized as follows:

(1) Basic strategy: Select beam directions that yield larger variances in each host ICP and a smaller correlation coefficient between the host ICPs.

(2) Edge detection scheme: To implement guideline (1), an edge detection scheme based on DTCWT applied to ECP was proposed for selecting sampling points, as ECP measurements require significantly less time than ICP measurements.

(3) Dopant estimation accuracy: In addition to edge detection, dopant estimation accuracy can generally be improved by ensuring that regions near the zone axis constitute 20–30% of the total beam directions.

(4) Exception for substantial AFF differences: In cases where the dopant AFF differs substantially from the host AFFs, following guideline (3) may lead to lower accuracies; hence, sampling from the near zone axis area should be avoided.

We verified that these sampling guidelines can reduce total measurement time by at least two orders of magnitude while maintaining occupancy accuracies comparable to those achieved with full beam tilts. These strategies also help minimize electron irradiation damage. Furthermore, the time saved by employing the proposed scheme can be used to improve data SNR by significantly increasing the data acquisition time per tilt for trace dopants.

The effective sampling presented in this study was achieved by digitally controlling the electron probe using a software plug-in for the Gatan Microscopy Suite platform. However, this method of beam control remains a challenge for reducing total measurement time compared to hardware-controlled HARECXS. The total measurement time could be further reduced by an order of magnitude or more if the procedure was implemented in the ROM with support from a microscope provider.

## Supplementary Material

dfaf004_Supp

## Data Availability

The data are available upon reasonable request.

## Abbreviations

Ac-STEM, aberration-corrected scanning transmission electron microscope;

ALCHEMI, atom-location by channeling-enhanced microanalysis;

St-ALCHEMI, statistical ALCHEMI; AFF, absorptive form factor; ASID, attachment scanning image display; DTCWT, dual-tree complex wavelet transform; ECP, electron-channeling pattern;

EDS, energy-dispersive X-ray spectroscopy;

HARECXS, high-angular resolution electron channeled X-ray spectroscopy;

HARECES, high-angular resolution electron channeled electron spectroscopy;

ICP, ionization channeling pattern;

SD, standard deviation; SNR, signal-to-noise ratio
