# Supplementary material for: Efficient data sampling scheme to reduce acquisition time in statistical ALCHEMI
Source: Microscopy (Oxf). 2025 Jan 13;74(4):267–78. doi: 10.1093/jmicro/dfaf004 (PMC12342804; doi:10.1093/jmicro/dfaf004)
Supplement: dfaf004_Supp [file dfaf004_supp.zip › suppl_data/SI_20241217.docx]

**Supplementary Information**

**Efficient Data Sampling Scheme to Reduce Acquisition Time in Statistical ALCHEMI**

Akimitsu Ishizuka^1,2^*, Masahiro Ohtsuka^1,3^ and Shunsuke Muto^1,3^

^1^Department of Materials Physics, Graduate School of Engineering, Nagoya University, Chikusa-ku, Nagoya 464-8603, Japan, ^2^HREM Research Inc., 14-48 Matsukazedai, Higashimastuyama, 355-0055, Japan, ^3^Electron Nanoscopy Section, Advanced Measurement Technology Center, Institute of Materials and Systems for Sustainability, Nagoya University, Chikusa-ku, Nagoya 464-8603, Japan

* Correspondence should be addressed.

E-mail: aki@hremresearch.com

**S1. Dual-tree complex wavelet transform (DTCWT)**

*1) DTCWT for edge detection in an image using Matlab functions*

Fig. S1(a) shows a 2D disk with a 50-pixel radius, where the image intensity decreases from one to zero towards the disk center over five pixels in a 100 × 100 pixel image. Figure S1(b) shows the 2D image reconstructed using the dual-tree complex wavelet transform (DTCWT) with the ‘dualtree2’ and ‘idualtree2’ functions in Matlab™, adding details up to level 4 with 'LowpassGain' set to zero. DTCWT yields an almost direction-independent result. The profile along the arrow in Fig. S1(b) is shown in Fig. S1(c), where the extrema of the reconstructed image align closely with the sharp edges of the slope. The mathematical details of DTCWT are described in [1].


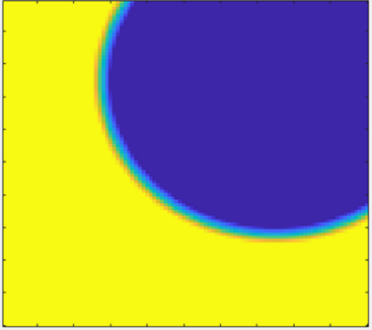

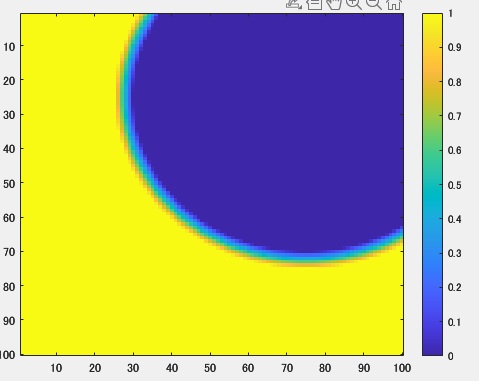

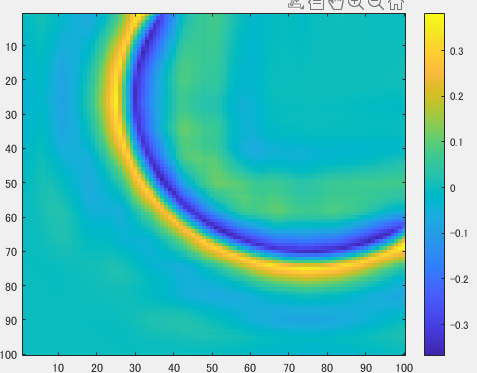

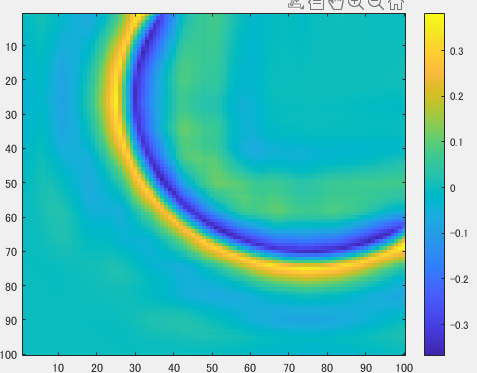

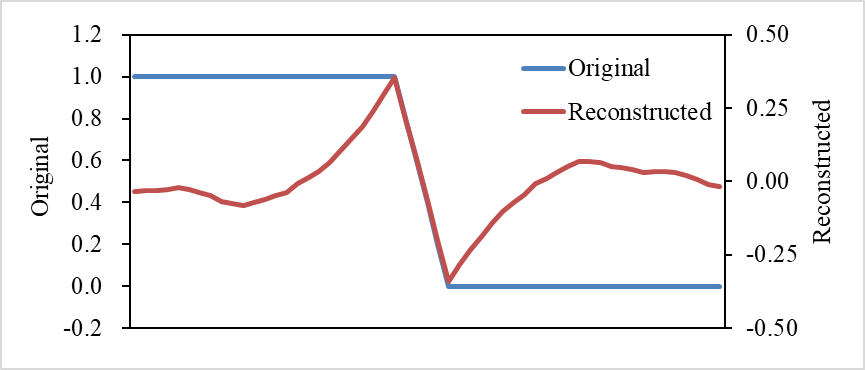


100 $\times$ 100 pixels^2^

**a**

**b**

**c**

**Fig. S1**. (a) Sample image of a disk with a sharp edge, where the intensity changes from 1 to 0 along the arrow. (b) Image reconstructed using DTCWT. (c) The line profiles of the original and reconstructed images along the arrows in (a) and (b).

*2) Typical DTCWT results for simulated data*

DTCWT was applied to a simulated ECP of Ca_2_SnO_4_ with dimensions 101 × 101 pixels (top left, Fig. S2). The other figures show the reconstructed images for varying the highest levels *J,* where the details up to level *J* were added up. The reconstructed image approaches the original image with increasing highest *J* level. The orange scale-bar in each reconstructed image indicates the length scale of the wavelet. Since the $2^{4}$ (= 16 pixels) scale is close to our edge detail, the highest level *J* of 4 was used with the setting ‘LowpassGain’ = 0.


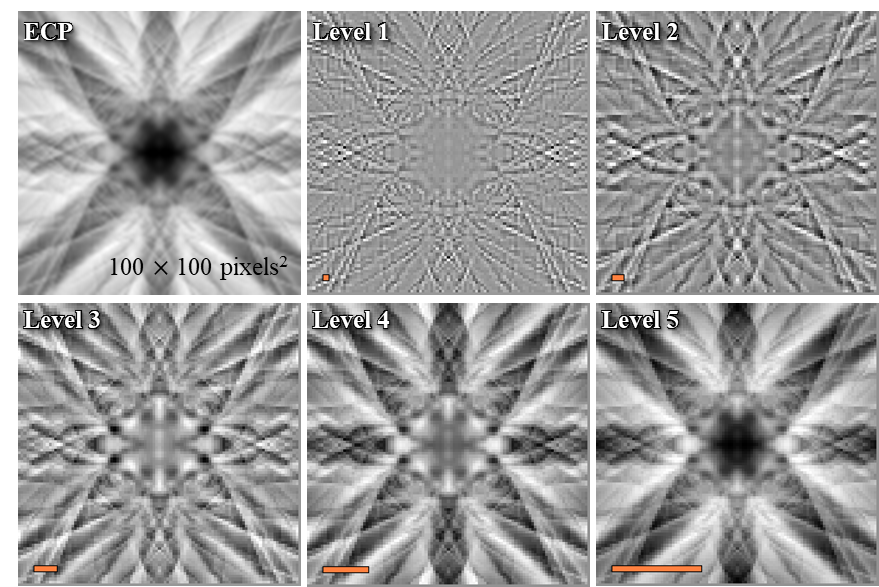


**Fig. S2**. Images reconstructed using DTCWT by changing the level *J*. The orange bars correspond to the wavelet support scale for each *J*.

**S2. Information of crystal structure**

*1)crystal structure of Ca_2_SnO_4_* [2]

Lattice Parameters: a: 5.75379 Å, b: 9.7016 Å, c: 3.26641 Å, $\alpha=\beta=\gamma:$90°

Space Group: Pbam [55]

Atom Coordinates

| Type | x | y | z |
| --- | --- | --- | --- |
| Ca | 0.0718 | 0.31768 | 0.5 |
| Sn | 0.0 | 0.0 | 0.0 |
| O | 0.3631 | 0.3097 | 0.0 |
| O | 0.2257 | 0.0502 | 0.5 |

*2)crystal structure of Y_2_Ti_2_O_7_* [2]

Lattice Parameters: a: 10.10 Å, b: 10.10 Å, c: 10.10 Å, $\alpha=\beta=\gamma:$90°

Space Group: Fd$\bar{3}$m [227]

Atom Coordinates

| Type | x | y | z |
| --- | --- | --- | --- |
| Y | 0.375 | 0.875 | 0.125 |
| Ti | 0.875 | 0.875 | 0.125 |
| O | 0.25 | 0.75 | 0.25 |
| O | 0.25 | 0.045075 | 0.25 |

**S3. Effect of adding the central part to edge detection simulation of during Y_2_Ti_2_O_7_ simulation**

The host ionization excitation probabilities for the incident beam directions near the zone axis are plotted in Fig. S3, with different colors in the order of their distance from the center of the zone axis. The Y intensity varies widely, whereas the variation in the Ti intensity was lower. In contrast to Ca_2_SnO_4_, the variances and correlation coefficients of the host intensities do not change significantly when these beam directions are included. Consequently, as presented in Table S1, the uncertainties in the dopant occupancy and fractional occupancy did not improve considerably when the incident beam directions close to the zone axis were included. Furthermore, the fraction deviated from the true value, and this tendency was more pronounced as the proportion of the central part increased. The reasons are explained in the Discussions section in the main text.


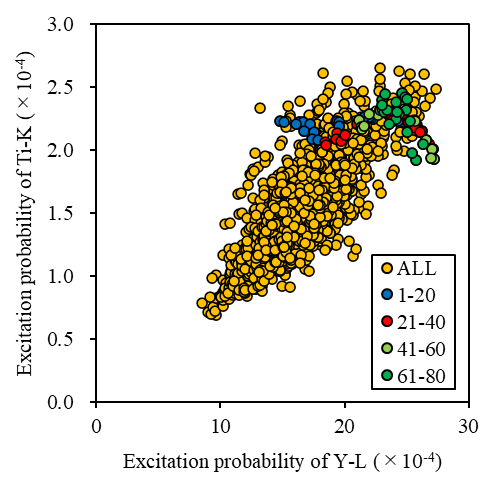


**Fig. S3.** Distribution of host ionization excitation probability for the incident beam directions near the zone axis. The beam directions are classified according to the distance from the zone-axis, and each of the 20 directions is color-coded.

**Table S1**. Occupancy, fractional occupancy, and variance $v_{i}$ and correlation coefficients $C_{Y,Ti}$ estimated from total 100 beam directions, including the center points. Results obtained via random selection are shown for comparison.

| Points from central region (%) | Occupancy (%) | | Fraction (%) | $v_{i}$ | | $C_{Y,Ti}$ |
| --- | --- | --- | --- | --- | --- | --- |
|  | Y site | Ti site | Y site | Y ($\times{10}^{-7}$) | Ti ($\times{10}^{-9}$) |  |
| 0 | 0.32±0.051 | 0.71±0.051 | 30.9±3.8 | 2.65 | 3.46 | 0.92 |
| 10 | 0.34±0.054 | 0.69±0.054 | 33.0±3.9 | 2.67 | 3.31 | 0.92 |
| 20 | 0.34±0.052 | 0.69±0.053 | 32.7±3.8 | 2.64 | 3.21 | 0.91 |
| 30 | 0.36±0.054 | 0.68±0.056 | 34.9±3.9 | 2.64 | 3.19 | 0.91 |
| 40 | 0.39±0.054 | 0.67±0.056 | 36.8±3.8 | 2.25 | 2.65 | 0.89 |
| 50 | 0.37±0.052 | 0.69±0.056 | 34.9±3.7 | 2.06 | 2.27 | 0.86 |
| Random | 0.34±0.059 | 0.70±0.058 | 32.8±4.3 | 1.09 | 1.47 | 0.83 |

**S4. DigitalMicrograph demo-script for the efficient data sampling scheme proposed in this study**

The ALCHEMI mode in the QED plugin [10-12] supports sequential data collection across equally spaced beam-tilt directions within line, square, or circular regions. However, our advanced data sampling scheme requires non-equally spaced beam-tilt directions targeting specific sampling points, which the existing ALCHEMI mode does not fully support.

To address this challenge, we have developed additional user-callable functions and integrated them into the QED plugin. These functions, summarized within a DigitalMicrograph (DM) script class named ‘QED_Publishing_class’, provides flexible control over beam-tilting while simultaneously correcting illumination aberrations. This enhancement is offered as a separate feature from the user-friendly ALCHEMI mode [12].

The ‘QED_Publishing_class’ includes the following functions:

1. ‘QEDP_setFrags’: Configures key parameters and fragments for data collection.

2. ‘QEDP_SaveOriginalBeamSettings’: Saves current beam settings allowing for potential reversion.

3. ‘QEDP_BeamTilt’: Allows for arbitrary beam-tilting according to user-defined directions for precise data sampling.

4. ‘QEDP_ResetBeamSettings’: Restores the original beam settings after data collection or adjustments.

5. ‘QEDP_Check_Beam’: Verifies the current beam settings to ensure alignment and accuracy before data collection.

Each of these functions is detailed in the accompanying code comments, providing guidance on their integration into the data collection workflow.

| Class QED_Publishing_class  {  object QEDP_setFrags(object self, Number fIllAberration, Number fDescan, Number nonLinDescan)  // Sets the flag whether to apply aberration correction and descan during beam tilt processing.  object QEDP_SaveOriginalBeamSettings(object self)  // Saves the original coil values.  Number QEDP_BeamTilt(object self, Number tiltX, Number tiltY)  // Performs beam tilt via QED. Unit of tiltX and tiltY are in mrad.  void QEDP_ResetBeamSettings(object self)  // Resets all coils to the values ​​saved with the QEDP_SaveOriginalBeamSettings function.  void QEDP_Check_Beam(object self)  // Display the settings for each coil in the results window.  } |
| --- |

Sequential beam-tilting over a user-defined incident beam directions can be achieved using the functions in the ‘QED_Publishing_class’ of the DigitalMicrograph (DM) script, as demonstrated in the following pseudocode:

| Object QED_P = alloc(QED_Publishing_class).init();  QED_P.QEDP_setFrags(fIllShift, fDescan, fNLDesctan);  QED_P.QEDP_SaveOriginalBeamSettings();  for{  QED_P.QEDP_BeamTilt(nTiltXmrad, nTiltYmrad); // Set tilting angle with mrad.  //Do anything you want.  }  QED_P.QEDP_ResetBeamSettings(); |
| --- |

In our St-ALCHEMI analysis using the efficient data sampling scheme, significant sampling points are first selected from an electron channeling pattern (ECP), as detailed in Sections 3.2.2 and 3.2.3. Subsequently, sequential EDS spectral data is collected over the predetermined beam directions. The following sample DM script was used for our experimental data collection. In this script, the variable ‘imgP’ should store a list of the predetermined incident beam tilting angles (*θ_x_*, *θ_y_*) along the *X*- and *Y*-directions. Notably, the image size of ‘imgP’ represents 2 × *n* pixels^2^, where *n* is the number of beam-tilt directions. The *X*-axis pixels represent the beam-tilting angles *θ_x_* and *θ_y_*, respectively. EDS spectra are then sequentially collected according to the beam-tilting conditions specified in ‘imgP’, resulting in sparse ICPs based on our efficient data sampling scheme."

| // $BACKGROUND$  // Acquisition parameters  Number nEDSCh = 4096; // EDS channel  Number nEDSDisp = 10; // dispersion eV/channel  Number nEDSAcqTime = 10; // acquisition time (in sec)  Number nDelay = 0.1; // delay after beam control (in sec)  // Main  Number nW,nH;  Image imgP; // The image to store a list of beam directions determined from the ECP  if(!GetOneImage("QED Tilting Parameter",imgP)) exit(0);  imgP.GetSize(nW, nH);  if(nW < 2) Exit(0);  Object QED_P = alloc(QED_Publishing_class).init(); // The instance QED_P is created here.  QED_P.QEDP_setFrags(1,0,0); // Sets some flags to the instance QED_P  QED_P.QEDP_SaveOriginalBeamSettings(); // Saves current beam settings  Number nTiltXmrad, nTiltYmrad;  Image imgOut := RealImage("ALCHEMI Stack", 4, nEDSCh, nH);  Image imgDisp := EDSAcquireSpectrum(nEDSCh, nEDSDisp, nEDSAcqTime);  Number origin,scale,calibrationFormat;  String units;  imgDisp.ImageGetDimensionCalibration(0, origin, scale, units, calibrationFormat);  imgOut.ImageSetDimensionCalibration(0, origin, scale, units, calibrationFormat);  showimage(imgDisp);  try{  for(number i;i<nH;i++){  nTiltXmrad = imgP.GetPixel(0, i); // Get the X-beam tilting angle from the list  nTiltYmrad = imgP.GetPixel(1, i); // Get the Y-beam tilting angle from the list  QED_P.QEDP_BeamTilt(nTiltXmrad,nTiltYmrad) // Set beam tilting angle  sleep(nDelay);  imgOut[i,0,i+1,nEDSCh] = EDSAcquireSpectrum(nEDSCh, nEDSDisp, nEDSAcqTime);  if(ShiftDown()){  if(OkCancelDialog("Stop Acquiring?")){  break;  }  }  }  }  recover{  showimage(imgOut);  QED_P.QEDP_ResetBeamSettings(); // Restore beam settings  } |
| --- |

**References**

[1] Ivan W. Selesnick, Richard G. Baraniuk, and Nick G. Kingsbury: IEEE Signal Processing Magazine, Nov. 2005, pp. 123-151

[2] https://discover.materialscloud.org/topomat/materials/3804/

[3] https://next-gen.materialsproject.org/materials/mp-5373
